# Supplementary material for: Ubiquitination of RIPK1 suppresses programmed cell death by regulating RIPK1 kinase activation during embryogenesis
Source: Nat Commun. 2019 Sep 13;10:4158. doi: 10.1038/s41467-019-11839-w (PMC6744433; doi:10.1038/s41467-019-11839-w)
Supplement: Supplementary file 1 — Supplementary Information [file 41467_2019_11839_MOESM1_ESM.pdf]

Supplemental Information

Ubiquitination of RIPK1 suppresses programmed cell death by regulating RIPK1 kinase activation during embryogenesis

Zhang *et al.*

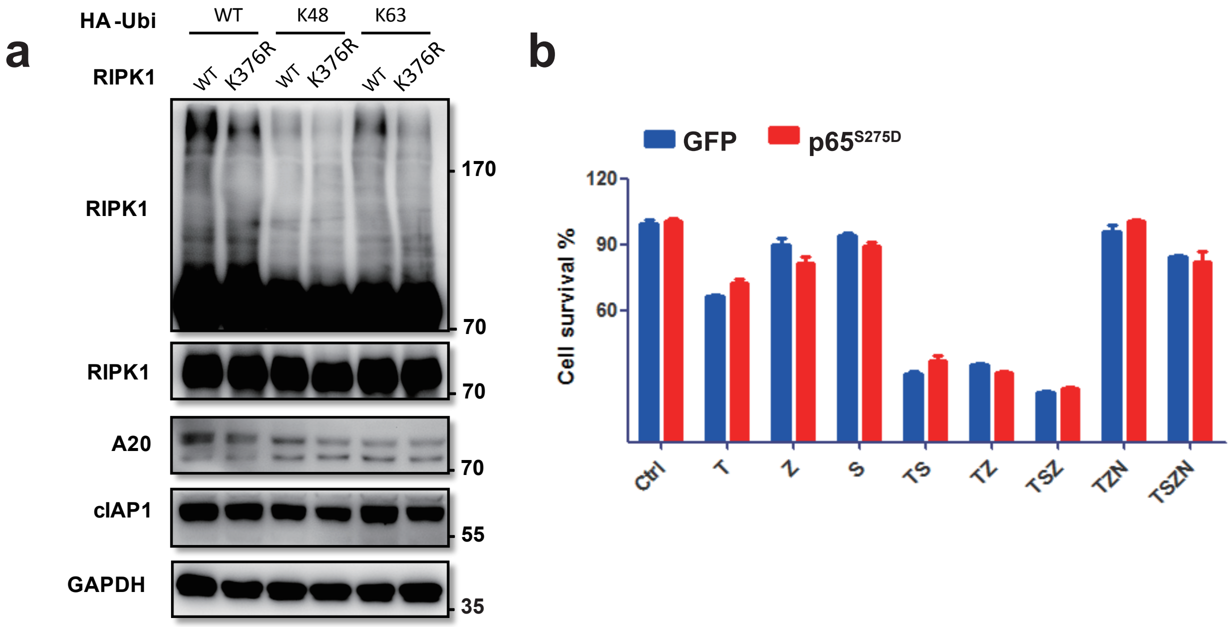

Supplementary Figure. 1 Defects of K63-mediated RIPK1 ubiquitination induced cell death is independent on impaired NF-κB pathway. Related to Fig. 2

a HEK293T cells were transfected with vectors expressing HA-Ub and RIPK1 or RIPK1^{K376R}. The ubiquitinated RIPK1 was enriched by HA immunoprecipitation and detected using aniti-RIPK1 antibody. **b** Ripk1$^{K376R/K376R}$ MEFs infected with lentivirus to stably expressing GFP or p65^{S275D} were treated with indicated drugs for 24 hours. Cell viability was determined using the CellTiter-Glo kit. The data are represented as the mean ± SEM of three independent experiments. Abbreviations are as follows: Ctrl, untreated; T, TNF-α (20ng/ml); S, Smac mimetic(100nM); Z, zVAD(20μM); TS, TNF-α (20ng/ml)+Smac mimetic(100nM); TZ,TNF-α(20ng/ml)+zVAD(20μM); TSZ, TNF-α (20ng/ml)+Smac mimetic(100nM)+zVAD(20μM); TZN, TNF-α+zVAD+Necrostatin-1(20μM); TSZN, TNF-α+Smac mimetic +zVAD+Necrostatin-1(20μM).Source data are provided as a Source Data file.

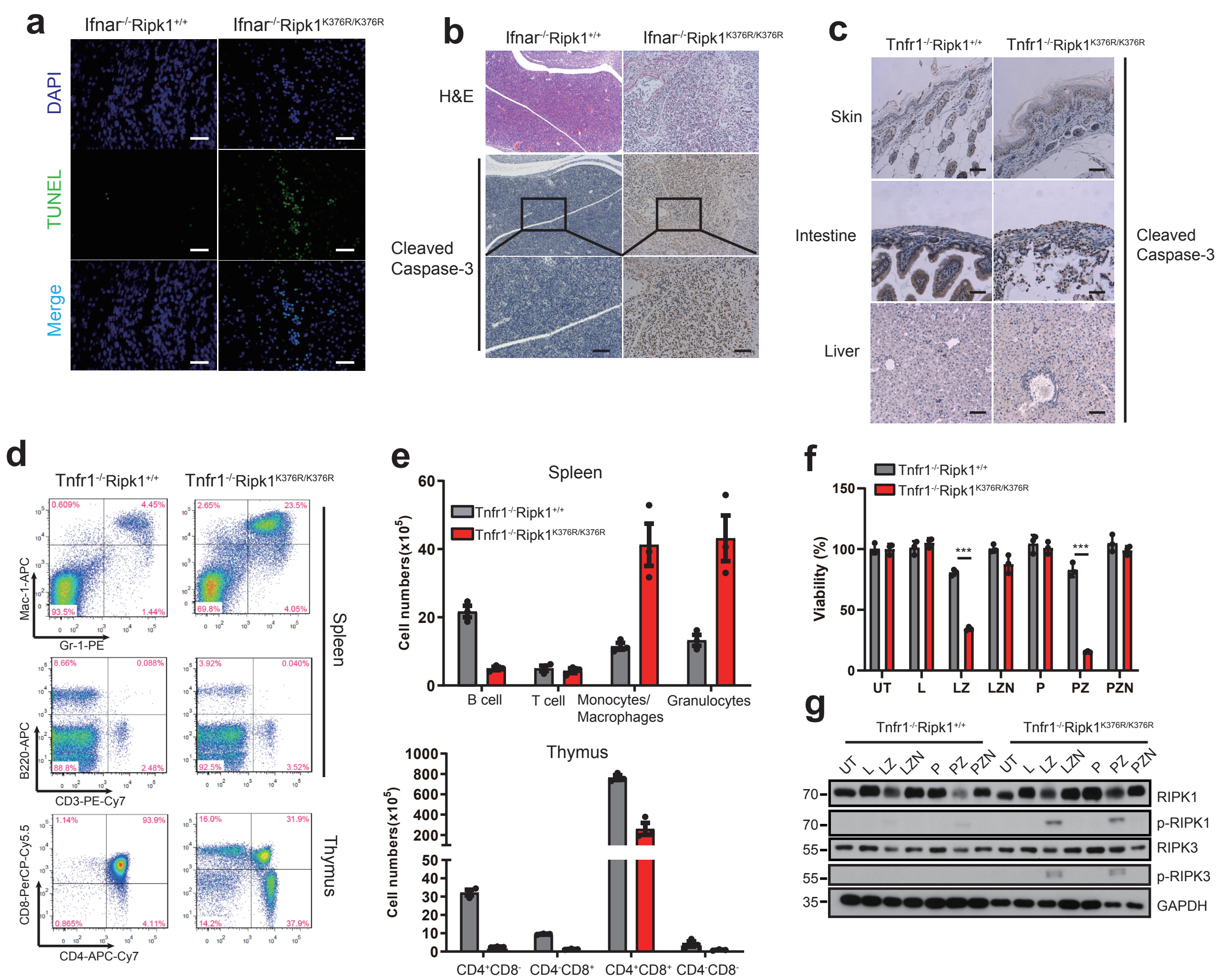

Supplementary Figure 2. Ablation of Ifnar does not prevent the embryonic lethality of Ripk1$^{K376R/K376R}$ mice. Related to Fig. 6

a TUNEL staining and **b** cleaved caspase-3 staining of fetal livers from mouse embryos with indicated genotypes. Scale bars, 50μm. **c** Cleaved caspase-3 stained tissues from Tnfr1$^{-/-}$Ripk1$^{K376R/K376R}$ and littermate control. **a-c** Results are representative of three mice of each genotype (Scale bars, 100μm). **d** Representative flow cytometric images of myeloid cells (Gr-1^{+}CD11b^{+}), B cells (B220^{+}) and T cells (CD3^{+}) in spleens and CD4^{+}, CD8^{+}, CD4^{+}CD8^{+} cells in thymus from 1-week-old Tnfr1$^{-/-}$ Ripk1$^{+/+}$ and Tnfr1$^{-/-}$ Ripk1$^{K376R/K376R}$ mice. **e** Cell counts in spleen and thymus from 1-week-old mice of indicated genotypes. Data are represented as mean ± SEM (n=3/genotype). **f** BMDMs were isolated from Tnfr1$^{-/-}$ Ripk1$^{+/+}$ and Tnfr1$^{-/-}$Ripk1$^{K376R/K376R}$ mice and were treated with L, LZ, LZN, PZ and PZN for 6h, respectively. Cell viability was determined using the CellTiter-Glo kit. Data are represented as mean ± SEM of BMDMs derived from three mice. P value was determined by Student's t-test (***p<0.001). Abbreviations are as follows: UT, untreated; LZ, LPS (20ng/ml)+zVAD(20μM); LZN, LPS+zVAD+Nec-1(20μM); PZ, Poly(I:C) (50ug/ml)+zVAD; PZN, Poly(I:C)+zVAD+Nec-1. **g** Tnfr1$^{-/-}$ Ripk1$^{+/+}$ and Tnfr1$^{-/-}$Ripk1$^{K376R/K376R}$ BMDMs were treated with LZ and PZ for 3h with or without Nec-1s. Immunoblottings of indicated proteins were shown.

a

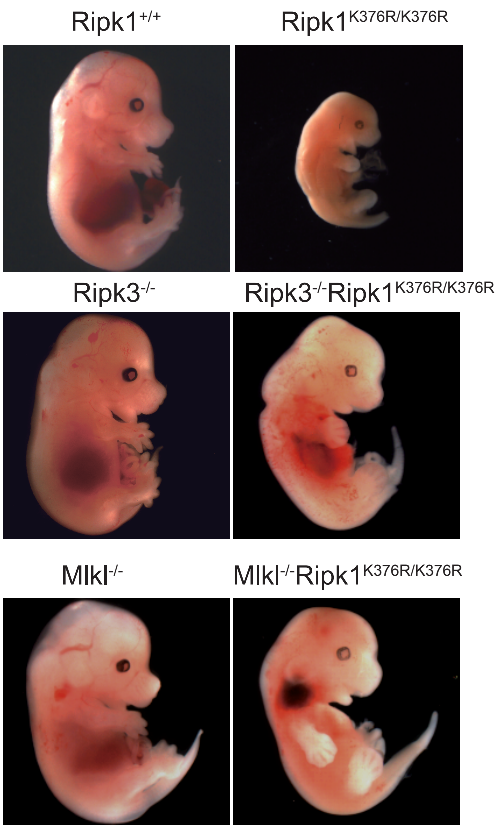

b

Ripk3$^{-/-}$Ripk1$^{K376R/+}$ intercrossing

Stage	Ripk3$^{-/-}$ Ripk1$^{+/+}$	Ripk3$^{-/-}$ Ripk1$^{K376R/+}$	Ripk3$^{-/-}$ Ripk1$^{K376R/K376R}$	Total
E13.5	4	13	6	23
E14.5	6	10	12	28
E15.5	9	17	2 *	28
>P0	12	18	0	30

Mlkl$^{-/-}$Ripk1$^{K376R/+}$ intercrossing

Stage	Mlkl$^{-/-}$ Ripk1$^{+/+}$	Mlkl$^{-/-}$ Ripk1$^{K376R/+}$	Mlkl$^{-/-}$ Ripk1$^{K376R/K376R}$	Total
E13.5	6	15	4	25
E14.5	7	15	8	30
E15.5	4	21	3 *	28
>P0	10	22	0	32

Supplementary Figure 3. Deletion of Ripk3 or Mlkl fails to prevent the embryonic lethality of Ripk1$^{K376R/K376R}$ mice. Related to Fig. 7

a Embryos of the indicted genotypes at E14.5. Images are representative of embryos indicated each genotypes (n=4-15/genotype). **b** Expected and observed frequency of genotypes in offspring at weaning from crosses of Ripk3$^{-/-}$Ripk1$^{K376R/+}$ (up) or Mlkl$^{-/-}$Ripk1$^{K376R/+}$ (down).

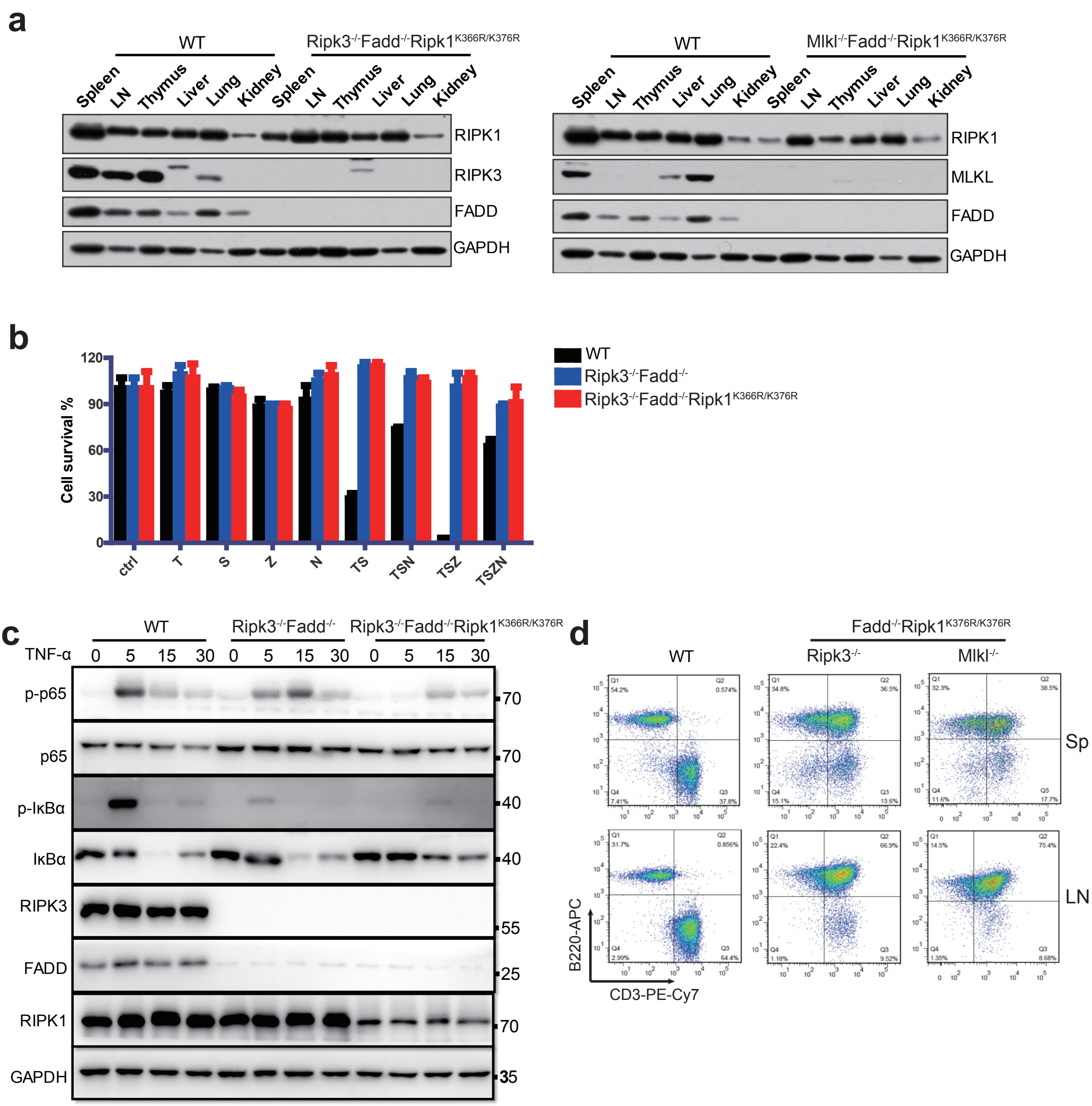

Supplementary Figure 4. Analysis of Ripk3$^{-/-}$Fadd$^{-/-}$Ripk1$^{K376R/K376R}$ and Mlkl$^{-/-}$Fadd$^{-/-}$Ripk1$^{K376R/K376R}$ mice. Related to Fig. 7

a Expression levels of the indicated protein in tissues from Ripk3$^{-/-}$Fadd$^{-/-}$ Ripk1$^{K376R/K376R}$ and Mlkl$^{-/-}$Fadd$^{-/-}$ Ripk1$^{K376R/K376R}$ mice. **b** Ripk1$^{+/+}$, Ripk3$^{-/-}$Fadd$^{-/-}$Ripk1$^{K376R/K376R}$ and Mlkl$^{-/-}$Fadd$^{-/-}$Ripk1$^{K376R/K376R}$ MEFs were treated with TS, TSN, TSZ, TSZN for 24h. Cell viability was determined using the CellTiter-Glo kit. The data are represented as the mean± SEM of three independent experiments. Abbreviations are as follows: Ctrl, untreated; T, TNF-α (20ng/ml); S, Smac mimetic (100nM); Z, zVAD (20μM); N, Necrostatin-1(20μM) **c** Ripk1$^{+/+}$, Ripk3$^{-/-}$Fadd$^{-/-}$ and Ripk3$^{-/-}$Fadd$^{-/-}$Ripk1$^{K376R/K376R}$ were treated with TNF-α (20ng/ml) for the indicated periods of time, NF-κB signaling pathway was examined by western blotting. **d** Representative flow cytometric images of cells stained with anti-CD3 and anti-B220 taken from 12-week-old animal of the indicated genotypes. At least three mice each genotype were analyzed independently with similar results.

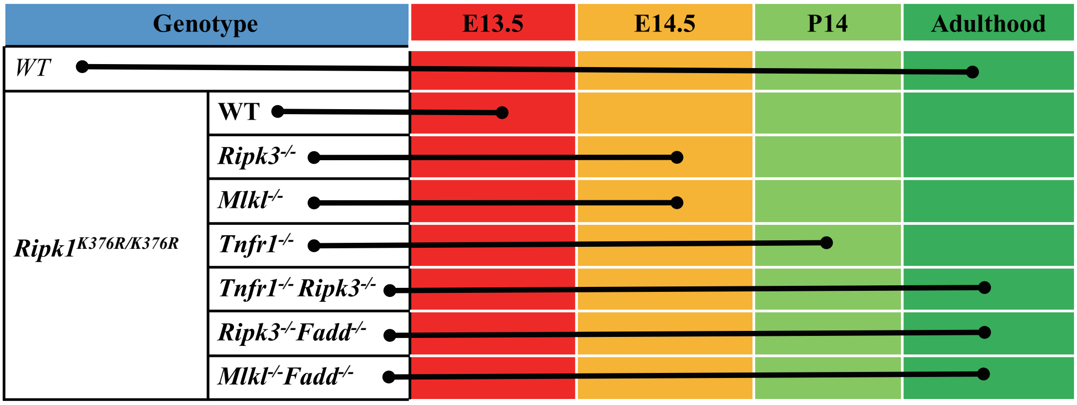

Supplementary Figure. 5 Genetic evidence highlights the role of RIPK1 ubiquitination on K376 during development. Related to Fig. 6,7

Genetic crosses of Ripk1$^{K376R/K376R}$mice with different combinations of gene deletions were shown

Supplementary Figure 6. Source data of the indicated Figures

Fig.1f

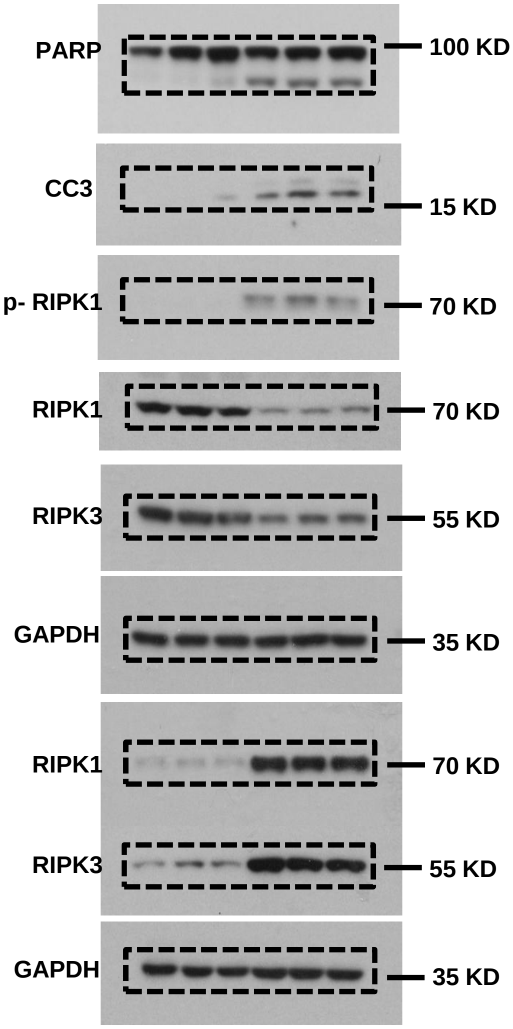

Fig.2a

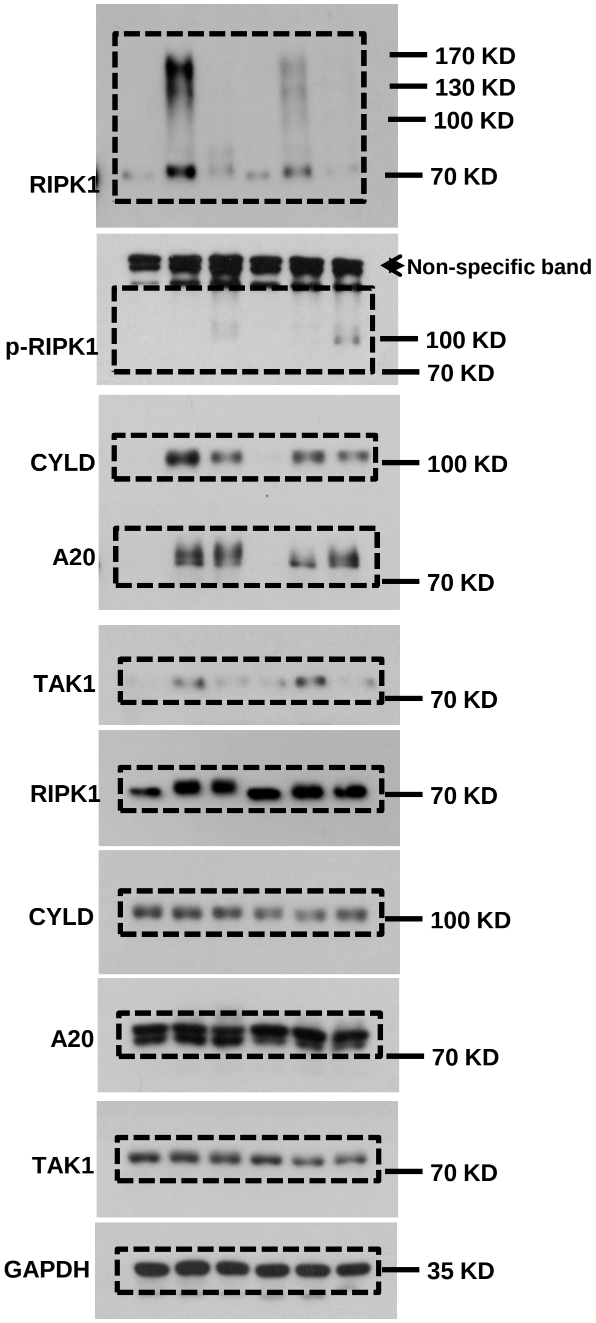

Fig.2b

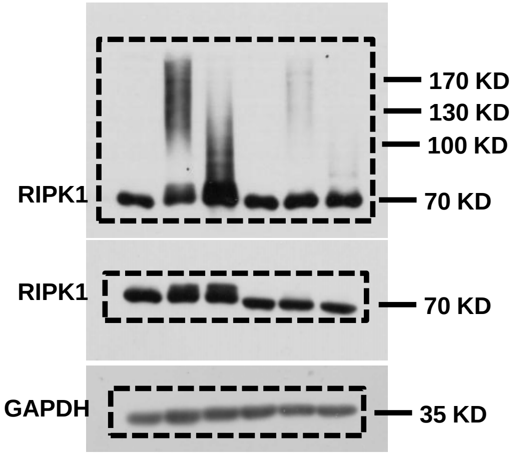

Fig.2c

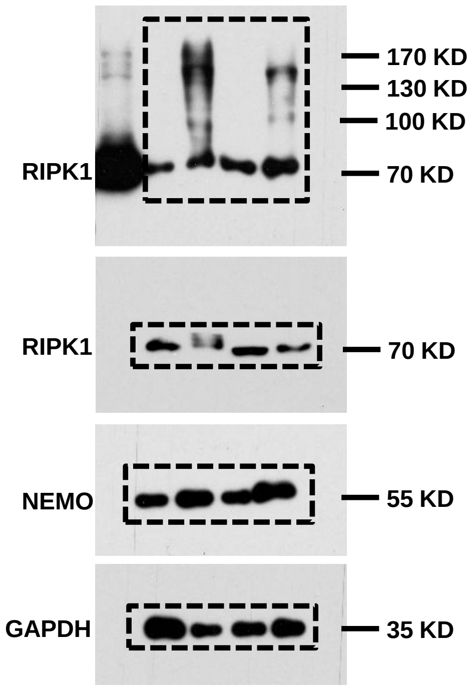

Fig.2d

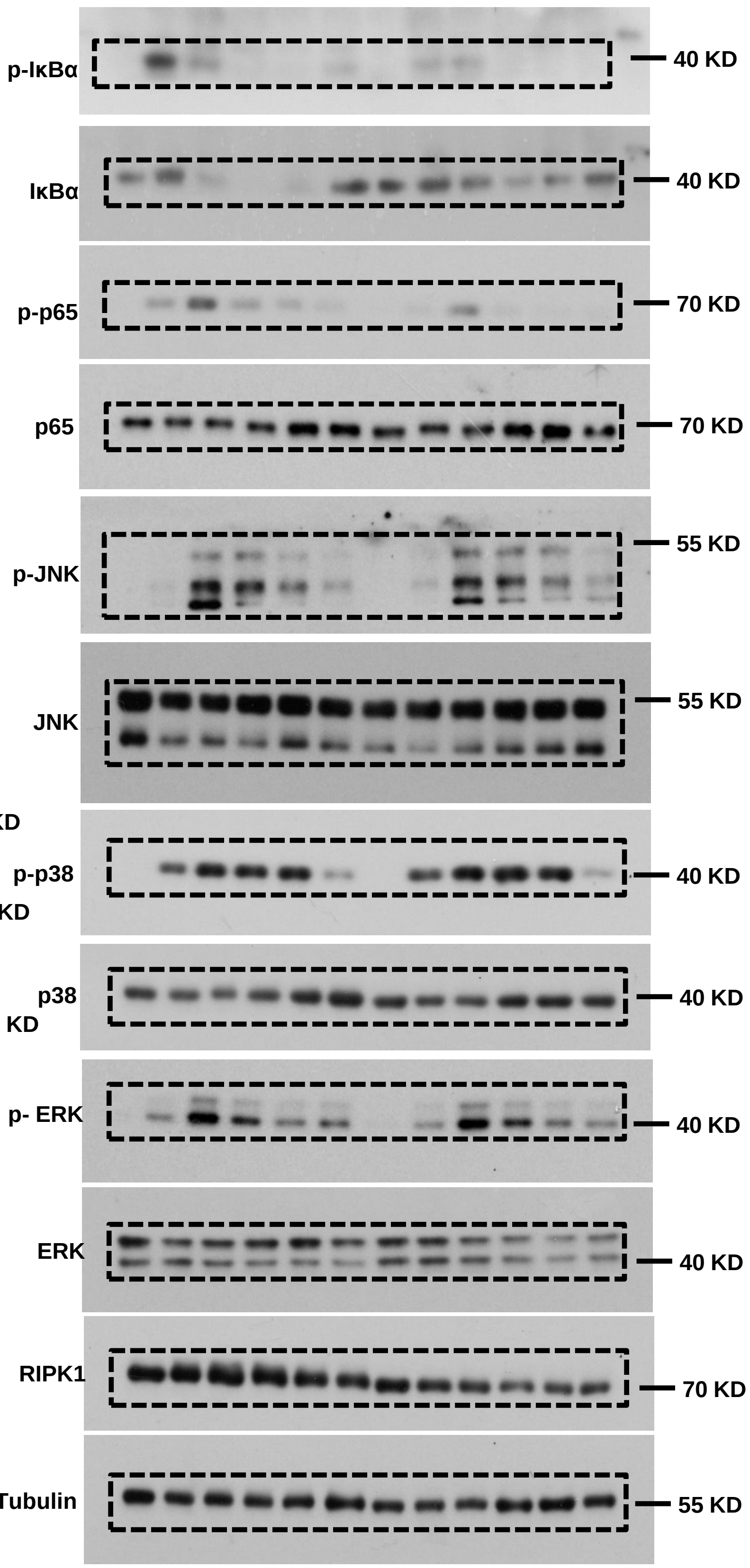

Fig.2e

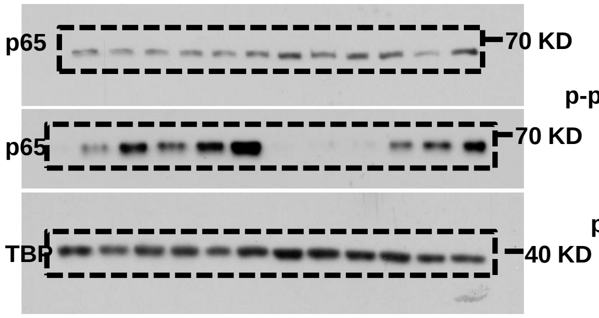

Fig.3b

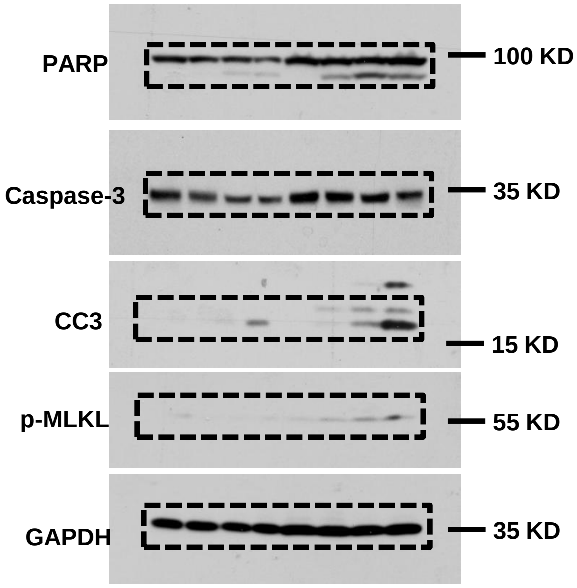

Fig.3c

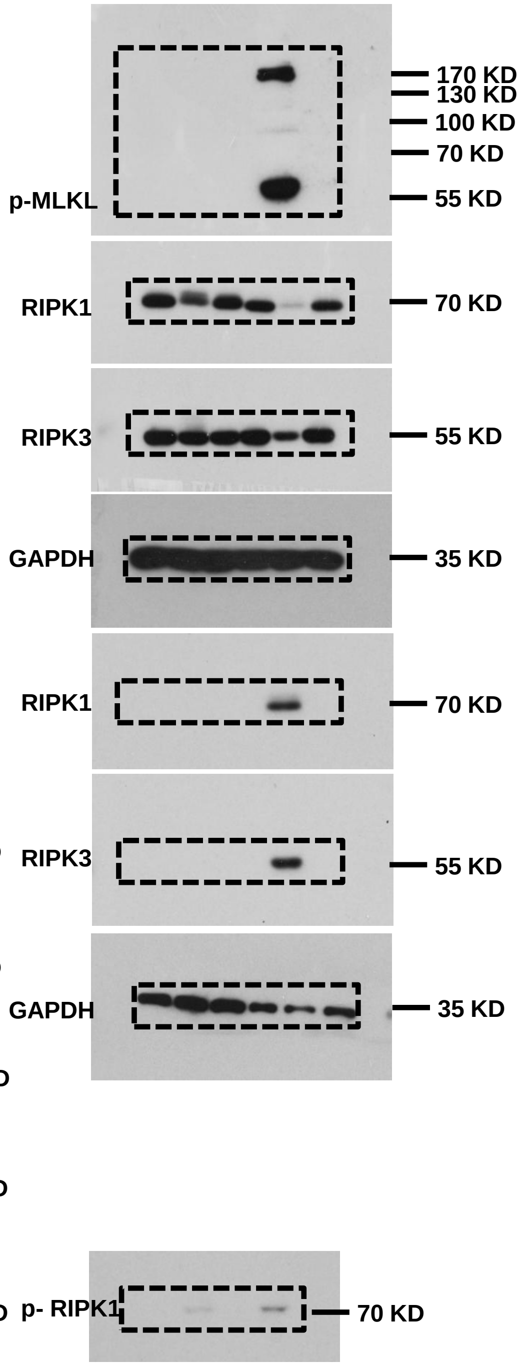

Fig.3d

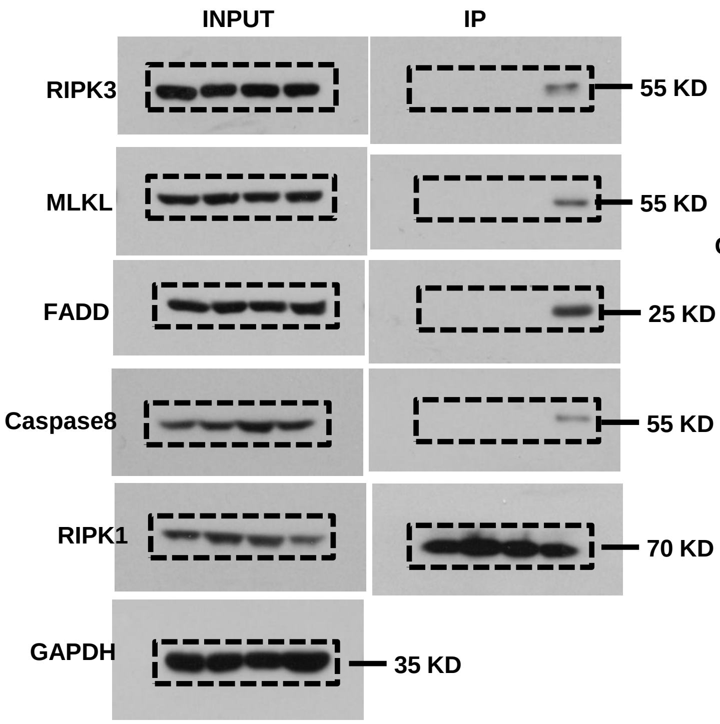

Fig.4f

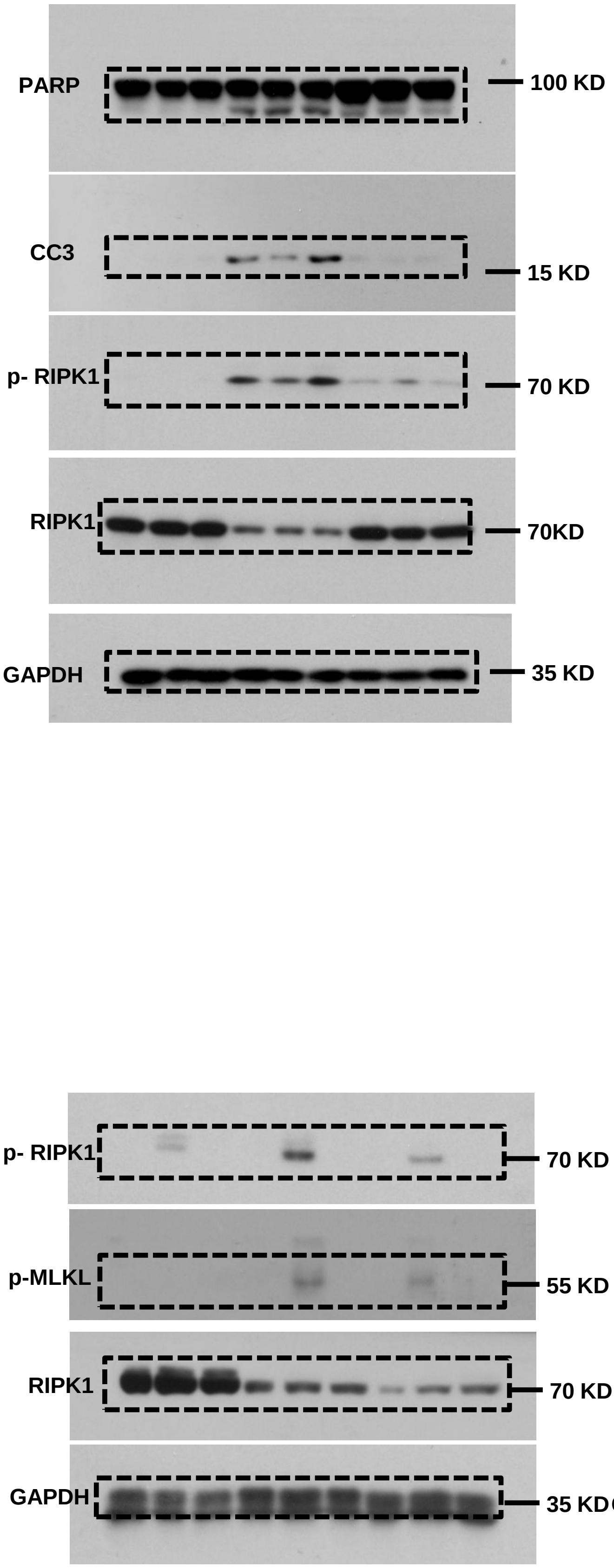
PARP
100 KD
CC3
15 KD
p- RIPK1
70 KD
RIPK1
70KD
GAPDH
35 KD
p- RIPK1
70 KD
p-MLKL
55 KD
RIPK1
70 KD
GAPDH
35 KD

Fig.5c

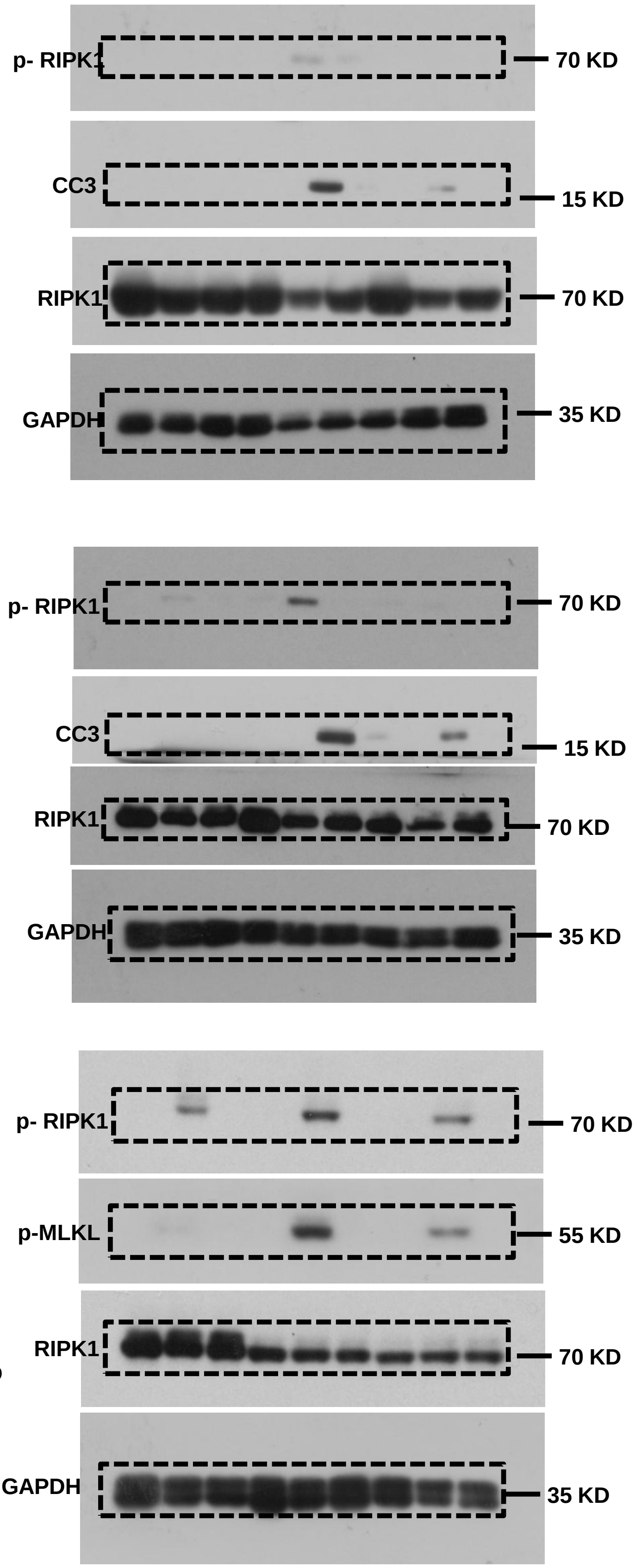
p- RIPK1
70 KD
CC3
15 KD
RIPK1
70 KD
GAPDH
35 KD
p- RIPK1
70 KD
CC3
15 KD
RIPK1
70 KD
GAPDH
35 KD
p- RIPK1
70 KD
p-MLKL
55 KD
RIPK1
70 KD
GAPDH
35 KD

Fig.S1a

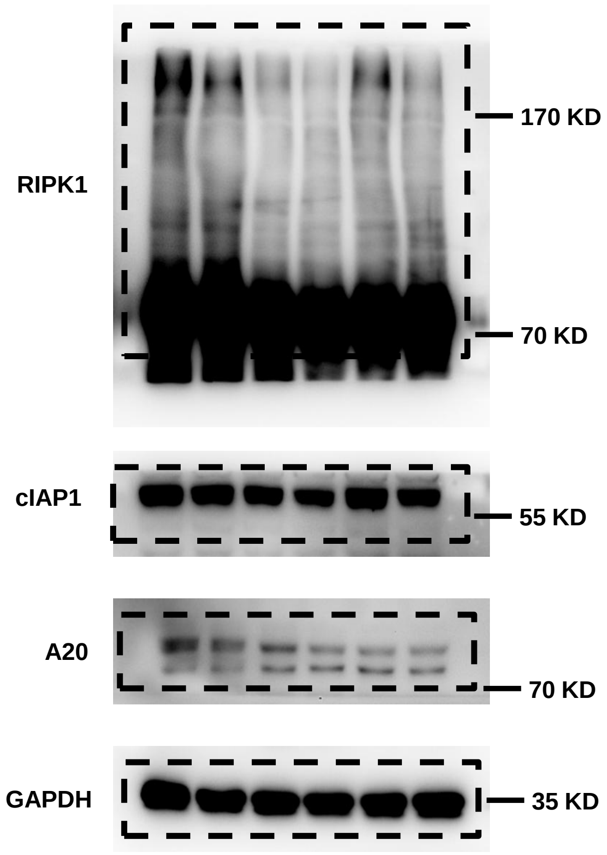

Fig.S2g

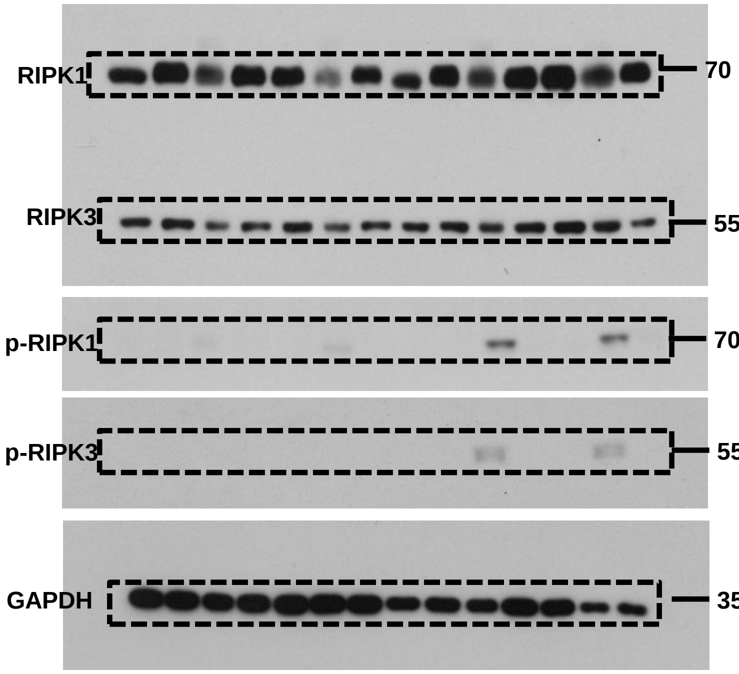

Fig.S4a

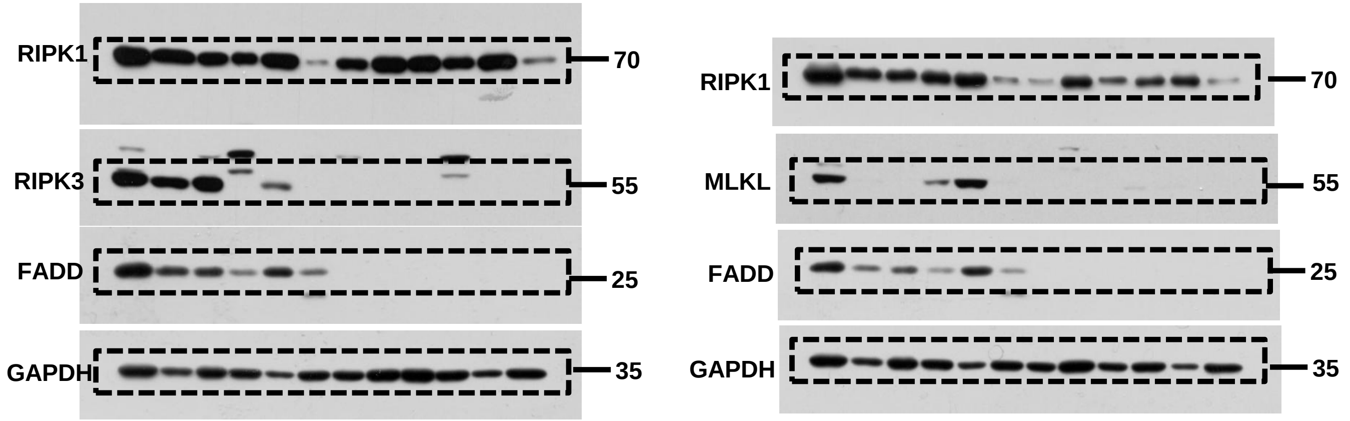

Fig.S4c

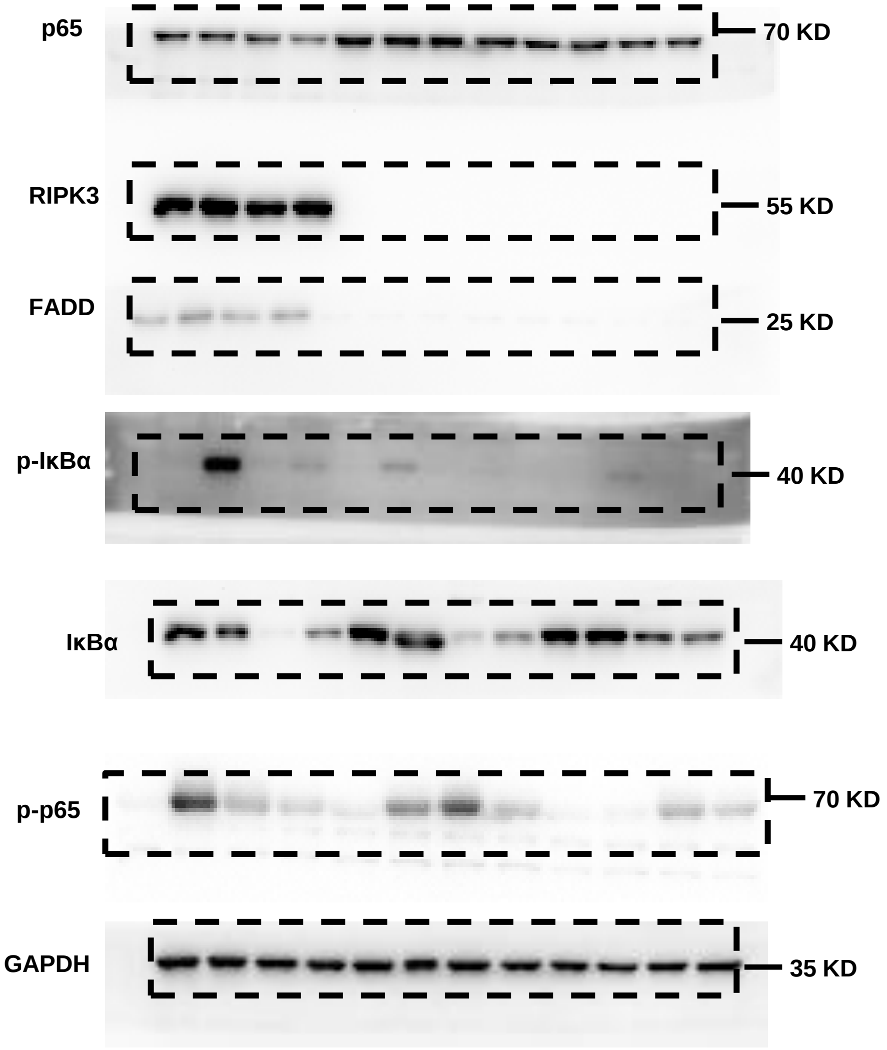
p65
70 KD
RIPK3
55 KD
FADD
25 KD
p-IκBα
40 KD
IκBα
40 KD
p-p65
70 KD
GAPDH
35 KD
