# Supplementary material for: Ubiquitination of RIPK1 suppresses programmed cell death by regulating RIPK1 kinase activation during embryogenesis
Source: Nat Commun. 2019 Sep 13;10:4158. doi: 10.1038/s41467-019-11839-w (PMC6744433; doi:10.1038/s41467-019-11839-w)
Supplement: Supplementary file 3 — Reporting Summary [file 41467_2019_11839_MOESM3_ESM.pdf]

nature research

Corresponding author(s): Haibing Zhang
Last updated by author(s): Jul 3, 2019

Reporting Summary

Nature Research wishes to improve the reproducibility of the work that we publish. This form provides structure for consistency and transparency in reporting. For further information on Nature Research policies, see Authors & Referees and the Editorial Policy Checklist.

Statistics

For all statistical analyses, confirm that the following items are present in the figure legend, table legend, main text, or Methods section.

n/a	Confirmed	
☐	☒	The exact sample size (*n*) for each experimental group/condition, given as a discrete number and unit of measurement
☐	☒	A statement on whether measurements were taken from distinct samples or whether the same sample was measured repeatedly
☐	☒	The statistical test(s) used AND whether they are one- or two-sided<br>*Only common tests should be described solely by name; describe more complex techniques in the Methods section.*
☒	☐	A description of all covariates tested
☒	☐	A description of any assumptions or corrections, such as tests of normality and adjustment for multiple comparisons
☐	☒	A full description of the statistical parameters including central tendency (e.g. means) or other basic estimates (e.g. regression coefficient) AND variation (e.g. standard deviation) or associated estimates of uncertainty (e.g. confidence intervals)
☒	☐	For null hypothesis testing, the test statistic (e.g. *F*, *t*, *r*) with confidence intervals, effect sizes, degrees of freedom and *P* value noted<br>*Give P values as exact values whenever suitable.*
☒	☐	For Bayesian analysis, information on the choice of priors and Markov chain Monte Carlo settings
☒	☐	For hierarchical and complex designs, identification of the appropriate level for tests and full reporting of outcomes
☒	☐	Estimates of effect sizes (e.g. Cohen's *d*, Pearson's *r*), indicating how they were calculated

Our web collection on statistics for biologists contains articles on many of the points above.

Software and code

Policy information about availability of computer code

Data collection	*Provide a description of all commercial, open source and custom code used to collect the data in this study, specifying the version used OR state that no software was used.*
Data analysis	GraphPad Prism 8, Microsoft Office Excel

For manuscripts utilizing custom algorithms or software that are central to the research but not yet described in published literature, software must be made available to editors/reviewers. We strongly encourage code deposition in a community repository (e.g. GitHub). See the Nature Research guidelines for submitting code & software for further information.

Data

Policy information about availability of data

All manuscripts must include a data availability statement. This statement should provide the following information, where applicable:

- Accession codes, unique identifiers, or web links for publicly available datasets
- A list of figures that have associated raw data
- A description of any restrictions on data availability

All data presented in this study are available within the Figures and its supplementary information file. Other data that support the study are available from the corresponding author upon reasonable request.

Field-specific reporting

Please select the one below that is the best fit for your research. If you are not sure, read the appropriate sections before making your selection.

- [x] Life sciences
- [ ] Behavioural & social sciences
- [ ] Ecological, evolutionary & environmental sciences

For a reference copy of the document with all sections, see nature.com/documents/nr-reporting-summary-flat.pdf

Life sciences study design

All studies must disclose on these points even when the disclosure is negative.

Sample size	All experiments were done with at least three biological repicates per group
Data exclusions	No data were excluded
Replication	All phenotypical data were representative of at least three independent biological samples and functional experiments were repeated at three times to draw a conclusion.
Randomization	Mice were randomly assigned to experiments disregarding their sex.
Blinding	Blinding was not used.

Reporting for specific materials, systems and methods

We require information from authors about some types of materials, experimental systems and methods used in many studies. Here, indicate whether each material, system or method listed is relevant to your study. If you are not sure if a list item applies to your research, read the appropriate section before selecting a response.

Materials & experimental systems

n/a	Involved in the study	
[ ]	[x]	Antibodies
[ ]	[x]	Eukaryotic cell lines
[x]	[ ]	Palaeontology
[ ]	[x]	Animals and other organisms
[x]	[ ]	Human research participants
[x]	[ ]	Clinical data

Methods

n/a	Involved in the study	
[x]	[ ]	ChIP-seq
[ ]	[x]	Flow cytometry
[x]	[ ]	MRI-based neuroimaging

Antibodies

Antibodies used	The following antibodies were used for western blotting and immunoprecipitation experiments: RIPK1(BD Biosciences,610459), RIPK1 (Cell Signaling Technology, 3493P),p-RIPK1(a gift from Junying Yuan's lab), RIPK3 (Prosci,2283), p-RIPK3(Abcam, ab195117), Caspase-8(Enzo Life Science,ALX-804-447-C100), MLKL (Abgent, ap14272b), p-MLKL (Abcam, ab196436), ?-actin(Sigma, A3854), Tublin(Sigma, T6199), IkBα(Cell Signaling Technology, 9242S), p-IkBα(Cell Signaling Technology,9246S), p-ERK(Cell Signaling Technology,9101S), ERK(Cell Signaling Technology,9102S), p-p38 (Cell Signaling Technology,9211S), p38(Cell Signaling Technology,9228S), p-p65(Cell Signaling Technology,3033S), p65(Cell Signaling Technology,4764S), p-JNK(Cell Signaling Technology,9251S), JNK(Cell Signaling Technology,9252S), PARP(Cell Signaling Technology ,9542S), Caspase-3(Cell Signaling Technology,9662S),TBP(Cell Signaling Technology ,44059S), FADD antibody was from Dr.Jianke Zhang's lab (Thomas Jefferson University).Antibodies against mouse CD3, CD4, CD8, B220, Gr-1, CD11b were purchased from eBioscience)
Validation	Most antibodies were from commercial sources. Anti-phosphorylated RIPK1 was a gift from Dr. Junying Yuan's lab and had been validated in published articles. Anti-FADD was obtained from Dr. Jianke Zhang's lab and had been validated in published articles

Eukaryotic cell lines

Policy information about cell lines

Cell line source(s)	Mouse Embryonic Fibroblasts (MEFs)
Authentication	MEFs were routinely examined by morphology.
Mycoplasma contamination	MEFs were regularly tested and confirmed to mycoplasma free.

Commonly misidentified lines (See ICLAC register)	No misidentified lines were used.

Animals and other organisms

Policy information about studies involving animals; ARRIVE guidelines recommended for reporting animal research

Laboratory animals	Mice used for experiments were all in C57BL6/J background.
Wild animals	This study did not involve wild animals.
Field-collected samples	This study did not involve field-collected samples.
Ethics oversight	Animal experiments were conducted in accordance with the guidelines of the Institutional Animal Care and Use Committee of the Institute of Nutrition and Health, Shanghai Institutes for Biological Sciences, University of Chinese Academy of Sciences.

Note that full information on the approval of the study protocol must also be provided in the manuscript.

Flow Cytometry

Plots

Confirm that:

- [x] The axis labels state the marker and fluorochrome used (e.g. CD4-FITC).
- [x] The axis scales are clearly visible. Include numbers along axes only for bottom left plot of group (a 'group' is an analysis of identical markers).
- [x] All plots are contour plots with outliers or pseudocolor plots.
- [x] A numerical value for number of cells or percentage (with statistics) is provided.

Methodology

Sample preparation	Lymphocytes from mouse spleen and LN were obtained by mashing through a cell strainer with a syringe plunger.
Instrument	FACS Aria III, BD biosciences
Software	FlowJo
Cell population abundance	Cell sorting was not used.
Gating strategy	For characterization of the lymphoid organs, cells were gated by FSC/SSC based on size and granularity for lymphocytes. B cells (CD19+ or B220+), T cells(CD3+), Monocytes and macrophages(CD11b+), Granulocytes(Gr-1+)<br>and B cell analysis) or myeloid cells (Mac1/Gr1 staining) were distinguished by individual cell surface marker.

- [ ] Tick this box to confirm that a figure exemplifying the gating strategy is provided in the Supplementary Information.
